# The university is (not) an ivory tower: an interview with Anna Schueth on breaking down barriers

**DOI:** 10.1038/s42003-023-04556-0

**Published:** 2023-02-20

## Abstract

Although we have come a long way in breaking down the stigma surrounding mental health conditions, we still have a long way to go. Here we speak to Dr Anna Schueth - a postdoc and passionate advocate whose blogs and other efforts are leading the way in changing academia towards a direction that will allow everyone to thrive as their authentic selves and to get the support they need.

**Figure:**
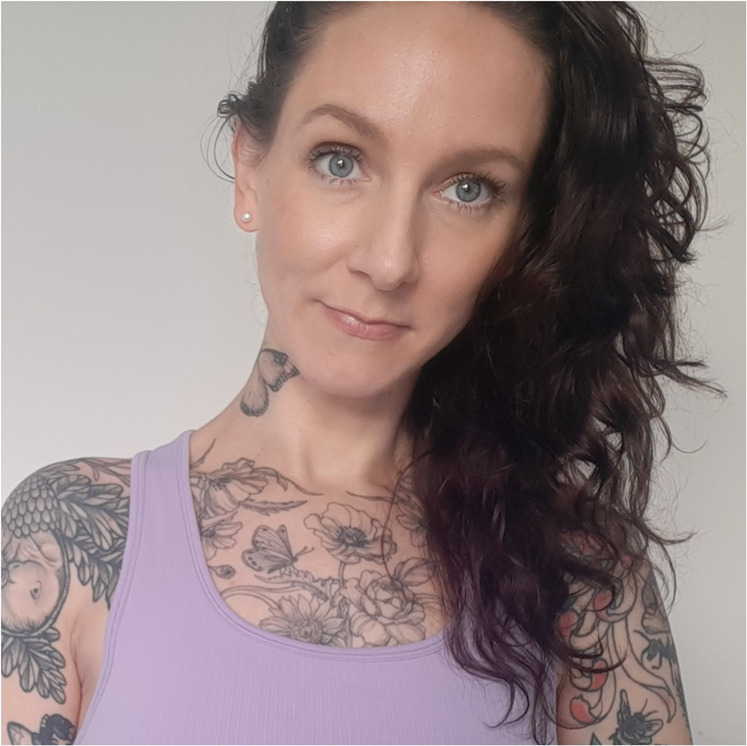
Anna Scheuth

*‘The university is (not) an ivory tower: with sharing academic mental health stories we can be role models and break down barriers’.*

Please tell us about yourself?

I am a postdoc at the Faculty of Neuroscience and Psychology, Maastricht University and work within the CBClab on large-scale, cleared tissue light-sheet imaging of post-mortem human brain and prostate cancer samples. As a recipient of the VENI research grant, which was awarded by the Dutch science organisation NWO, I have been working closely with local scientists, clinicians and international industrial partners on the co-development of a light-sheet microscope prototype. This contributes to our overall aim of advancing our understanding of 3D tissue morphology, such as prostate cancer patient tumours. In fact, our recent work has just been published in *Communications Biology*!

What has driven you to tackle mental health stigma in academia?

During the pandemic I came across so many students and staff members at my university who were suffering in silence. They disclosed to me very personal stories of suicidality, heart break, grief or being diagnosed with a mental health condition. Countless times I heard “Thank you for listening without judgement Anna. I am so glad I can share this with you and I am so scared to share that with anyone else, especially at work.” or “If someone finds out about my diagnosis, they will call me “crazy” and fire me. I have to put up a mask every day at work and pretend I am someone else. This is so hard.” I believe that struggles are part of life and that everyone has a story. However, oftentimes people in academia are scared to share their struggles, as they might be seen as weak or even fear losing their job. Therefore, I decided to take the first step and share my own mental health journey and personal struggles publicly on my blog for everyone to read. I contemplated doing this for a few weeks and discussed this with my family and friends, who all were supportive of this idea. “This will help so many and you can change people’s lives”. To this day I receive messages from people at my university and beyond who tell me that they feel less alone reading the blog posts that others and I wrote. Moreover, they were inspired to also share their stories in their academic work environment too and it makes me happy to see this positive snowball effect. Each and every person can contribute to the much needed change.

What can individuals do to support colleagues in academia?

In order to support our colleagues we have founded #FlourishMaastricht, which aims to reduce the academic mental health stigma across our local university and hospital communities. Together with Mark Kawakami from the Faculty of Law, we organised events and workshops to raise awareness, provide suicide prevention information (in collaboration with the Dutch Suicide Prevention Organization 113) and invite everyone to share their story in a safe space. It is a great sign that supervisors have reached out to us and asked how they can support their employees and students best. We would like to share information to increase mental health literacy and also provide practical advice, such as what to do, when someone deals with suicidality. Besides attending workshops and further sharing the information across all faculties, individuals can simply offer a listening ear to a colleague and check in once in a while. When we feel seen and safe, we are more comfortable speaking up.

What systemic change do you think is most needed?

In my opinion, it is crucial to create a safe and healthy work environment at the university, hospital and also outside of academia. We must be aware that we cannot just say “This is not happening at our university or within our team at work”. Many people and most likely also your direct colleagues within all communities are struggling with mental health conditions, which they may not disclose to anyone. Moreover, I know many who are dealing with a chronic illness, disability, their sexual identity, infertility, grief, loss, abuse, domestic violence, eating disorders or suicidality, which also greatly affects their mental health. It is important to realise that people are not just numbers in a system that have to produce output. I think only excellence is rewarded in academia and overworking is glamourised. This all leads to a toxic work culture with burned out individuals. We have to be and have better role models for work life balance - both for the first year students, as well as the PhD candidate. Furthermore, supervisors have to be mindful of deadlines, work pressure and workload. Employees should be encouraged to take their holidays and regularly switch off from work and not be applauded for working all year around. There are a number of students who have no choice but to have additional jobs alongside their studies and this causes a lot of extra pressure for them. Personally, I was dealing with this myself as a student and during this time I experienced a lot of despair, which resulted in a suicide attempt at the age of 20. Therefore, I can relate to this and understand how they feel. I would like to see more practical support for these students and in general everyone who is in a crisis situation. More emphasis has to be given to suicide prevention and awareness. I know people from all academic levels who have dealt with suicidal ideation or experienced a suicide attempt themselves. Sadly to this day, they do not feel comfortable sharing this publicly, as the stigma is still too high. I believe that we are on a good path, but more change in the academic work environment is needed (“Reducing the academic mental health stigma can save lives”, Nature Reviews Urology).

What advice would you give to someone struggling with their mental health in academia?

Everyone has personal and unique circumstances and there is no one fits all solution. It took me years to finally share something personal at work with my supervisor. During my PhD my father was very sick, had multiple surgeries and that affected me a lot. However, I kept this all to myself and did not want to show any weakness at work. Then one day he fell into a coma and that was the tip of the iceberg for me. I disclosed this to a colleague and then to my PhD supervisor at the time. My supervisor was extremely supportive and understanding. In hindsight this was a key moment for me and moving forward I started sharing personal traumatic experiences, such as the loss of my baby during my post doc with my current supervisor too. It was important for me to realise that is not a bad thing to share this and really important to ask for help. Therefore, my advice would be to speak with someone you trust. Ideally, this person (a colleague, your supervisor, a mentor or academic advisor) can listen to you and provide you with concrete advice, such as suggesting a therapist and/or professional help. Moreover, I would like to point that nothing is more important than your physical and mental health. I know that it is scary to ask for help and no matter how challenging your circumstances are, there are always solutions for this. It is crucial to never give up.

*This interview was conducted by Deputy Editor, Karli Montague-Cardoso*.

